# Rubella Virus Infected Macrophages and Neutrophils Define Patterns of Granulomatous Inflammation in Inborn and Acquired Errors of Immunity

**DOI:** 10.3389/fimmu.2021.796065

**Published:** 2021-12-20

**Authors:** Ludmila Perelygina, Raeesa Faisthalab, Emily Abernathy, Min-hsin Chen, LiJuan Hao, Lionel Bercovitch, Diana K. Bayer, Lenora M. Noroski, Michael T. Lam, Maria Pia Cicalese, Waleed Al-Herz, Arti Nanda, Joud Hajjar, Koen Vanden Driessche, Shari Schroven, Julie Leysen, Misha Rosenbach, Philipp Peters, Johannes Raedler, Michael H. Albert, Roshini S. Abraham, Hemalatha G. Rangarjan, David Buchbinder, Lisa Kobrynski, Anne Pham-Huy, Julie Dhossche, Charlotte Cunningham Rundles, Anna K. Meyer, Amy Theos, T. Prescott Atkinson, Amy Musiek, Mehdi Adeli, Ute Derichs, Christoph Walz, Renate Krüger, Horst von Bernuth, Christoph Klein, Joseph Icenogle, Fabian Hauck, Kathleen E. Sullivan

**Affiliations:** ^1^ Centers for Disease Control and Prevention, Division of Viral Diseases, Atlanta, GA, United States; ^2^ Department of Dermatology, Hasbro Children’s Hospital and Warren Alpert Medical School of Brown University, Providence, RI, United States; ^3^ Department of Pediatrics, University of Iowa Stead Family Children’s Hospital, Iowa City, IA, United States; ^4^ Department of Pediatrics, Texas Children’s Hospital, Baylor College of Medicine, Houston, TX, United States; ^5^ Pediatric Immunohematology and Bone Marrow Transplantation Unit and San Raffaele Telethon Institute for Gene Therapy (SR-TIGET), Istituto di Ricovero e Cura a Carattere Scientifico (National Institute for Research and Treatment) (IRCCS) San Raffaele Scientific Institute, Milan, Italy; ^6^ Department of Pediatrics, Kuwait University, Kuwait City, Kuwait; ^7^ Allergy and Clinical Immunology Unit, Department of Pediatrics, Al-Sabah Hospital, Kuwait City, Kuwait; ^8^ Pediatric Dermatology Unit, As’ad Al-Hamad Dermatology Center, Al-sabah Hospital, Kuwait City, Kuwait; ^9^ Department of Pediatrics, Queen Mathilde Mother and Child Centre, Antwerp University Hospital, Antwerp, Belgium; ^10^ Department of Dermatology, Queen Mathilde Mother and Child Centre, Antwerp University Hospital, Antwerp, Belgium; ^11^ Department of Dermatology, University of Pennsylvania Perelman School of Medicine, Philadelphia, PA, United States; ^12^ Department of Pediatrics, Dr. von Hauner Children’s Hospital, University Hospital, Ludwig-Maximilians-Universität München, Munich, Germany; ^13^ Department of Pathology and Laboratory Medicine, Nationwide Children’s Hospital, Columbus, OH, United States; ^14^ Department of Hematology, Oncology, Blood and Marrow Transplant, Nationwide Children’s Hospital, Columbus, OH, United States; ^15^ Department of Hematology, Children’s Hospital of Orange County, Orange, CA, United States; ^16^ Department of Pediatrics, University of California at Irvine, Orange, CA, United States; ^17^ Allergy/Immunology Section, Emory University, Atlanta, GA, United States; ^18^ Department of Pediatrics, University of Ottawa and Children’s Hospital of Eastern Ontario, Ottawa, ON, Canada; ^19^ Department of Dermatology, Oregon Health and Science University, Portland, OR, United States; ^20^ Division of Clinical Immunology, Department of Medicine, Icahn School of Medicine at Mount Sinai, New York, NY, United States; ^21^ Department of Pediatrics, National Jewish Health, Denver, CO, United States; ^22^ Department of Dermatology, University of Alabama at Birmingham, Birmingham, AL, United States; ^23^ Department of Pediatrics, University of Alabama at Birmingham, Birmingham, AL, United States; ^24^ Division of Dermatology, Washington University School of Medicine, St. Louis, MO, United States; ^25^ Division of Immunology and Allergy, Sidra Medicine and Hamad Medical Corporation, Doha, Qatar; ^26^ Center for Pediatric and Adolescent Medicine, University Medical Hospital Mainz, Mainz, Germany; ^27^ Institute of Pathology, Faculty of Medicine, Ludwig-Maximilians-Universität München, Munich, Germany; ^28^ Department of Pediatric Respiratory Medicine, Immunology and Critical Care Medicine, Charité – Universitätsmedizin Berlin, Berlin, Germany; ^29^ Charité - Universitätsmedizin Berlin, Corporate Member of Freie Universität Berlin, Humboldt-Universität zu Berlin, and Berlin Institute of Health (BIH), Berlin-Brandenburg Center for Regenerative Therapies (BCRT), Berlin, Germany; ^30^ Berlin Institute of Health at Charité – Universitätsmedizin Berlin, Berlin, Germany; ^31^ Labor Berlin GmbH, Department of Immunology, Berlin, Germany; ^32^ Division of Allergy Immunology, Department of Pediatrics, The Children’s Hospital of Philadelphia, University of Pennsylvania Perelman School of Medicine, Philadelphia, PA, United States

**Keywords:** inborn errors of immunity, primary immunodeficiency, vaccine-derived rubella viruses, granulomatous inflammation, skin lesion, neutrophils, macrophages, granuloma treatments

## Abstract

Rubella virus (RuV) has recently been found in association with granulomatous inflammation of the skin and several internal organs in patients with inborn errors of immunity (IEI). The cellular tropism and molecular mechanisms of RuV persistence and pathogenesis in select immunocompromised hosts are not clear. We provide clinical, immunological, virological, and histological data on a cohort of 28 patients with a broad spectrum of IEI and RuV-associated granulomas in skin and nine extracutaneous tissues to further delineate this relationship. Combined immunodeficiency was the most frequent diagnosis (67.8%) among patients. Patients with previously undocumented conditions, i.e., humoral immunodeficiencies, a secondary immunodeficiency, and a defect of innate immunity were identified as being susceptible to RuV-associated granulomas. Hematopoietic cell transplantation was the most successful treatment in this case series resulting in granuloma resolution; steroids, and TNF-α and IL-1R inhibitors were moderately effective. In addition to M2 macrophages, neutrophils were identified by immunohistochemical analysis as a novel cell type infected with RuV. Four patterns of RuV-associated granulomatous inflammation were classified based on the structural organization of granulomas and identity and location of cell types harboring RuV antigen. Identification of conditions that increase susceptibility to RuV-associated granulomas combined with structural characterization of the granulomas may lead to a better understanding of the pathogenesis of RuV-associated granulomas and discover new targets for therapeutic interventions.

## Introduction

Inborn errors of immunity (IEI) are a heterogeneous group of more than 450 monogenic disorders affecting different components of the immune system and manifesting with increased susceptibly to autoinflammation, autoimmunity, atopy, infection, bone marrow failure, and/or malignancy (1, 2). Chronic infection in patients with IEI can trigger formation of histopathological immune structures around antigens, resulting in granulomas, which primarily consist of macrophages and lymphocytes (3). If the immune system fails to clear the antigen, granulomas themselves can become a significant pathology with damage to the affected organ. The estimated prevalence of all types of granulomas (both sterile and non-sterile) in individuals with IEI is 1-4% (4). Effective treatment of granulomatous inflammation depends on correct identification of the combined immunological and microbial etiology, and often presents a diagnostic and therapeutic challenge for clinicians and pathologists (5).

Rubella virus (RuV) is a single-stranded positive sense RNA virus from the *Matonaviridae* family. Both wild type RuV and the live-attenuated vaccine strain RA27/3, which is part of the MMR vaccine used in most countries, can cause persistent infection resulting in several associated pathologies (6). Persistent infection of the developing fetus with wild type RuV often results in an array of developmental abnormalities known as congenital rubella syndrome (7, 8). Detection of RuV antigen in brain progenitor cells, alveolar macrophages, cardiac and vascular fibroblasts, the ciliary body of the eye, and in placental capillary endothelium correlate with organ abnormalities in this syndrome (9, 10). Other less common pathologies caused by persistent wild type RuV infection include rubella encephalitis, Fuchs’ uveitis, arthralgia, and arthritis (11–14).

Several cases of IEI with granuloma formation have recently been identified in association with RuV vaccine strain RA27/3 (15–20). Infectious immunodeficiency-related vaccine-derived rubella viruses (iVDRV) were isolated from granuloma biopsies and sequenced (16, 20). The iVDRV genomes contained multiple mutations which resulted in viruses with altered growth properties compared to the parental vaccine strain: iVDRV strains were less cytopathic, produced less infectious virus and could establish long-term persistent cultures in primary human fibroblasts (20). Patients with IEI develop rubella-associated granulomas from weeks to decades after MMR vaccination (18). The recent report of a patient with common variable immunodeficiency (CVID) with wild type RuV associated granulomas presenting in his 70s provides the first evidence that, in addition to vaccine virus strain, wild type RuV strains are also capable of long-term asymptomatic persistence and clinical re-emergence as symptomatic granulomas decades later (21). The cellular or tissue reservoir for latent iVDRV and wild type RuV, the mechanism of virus persistence, and the cause of virus-associated lesions in different organs is presently unknown. The risk factors and clinical manifestations remain incompletely described.

Cutaneous granulomas are the most frequent manifestation diagnosed, but other organs including the liver, spleen, and lungs can also be affected (4, 22, 23). RuV antigen is localized in M2 macrophages at the center of the granulomas in previously described cases (16, 17, 24, 25), but a detailed description of the spatial organization of RuV associated granulomas and its cellular constituents is lacking.

Here, we examine RuV-associated granulomas from 28 patients with inborn and acquired errors of immunity to determine whether specific inflammatory patterns localize to certain tissue sites or are associated with clinical characteristics. We present immunohistochemical evidence that macrophages and neutrophils play a role in defining RuV-associated granulomatous patterns in inflamed tissues. Serological and molecular data are analyzed to determine whether patients develop an efficient anti-rubella humoral immunity and whether RuV persisting in these patients are vaccine derived. The characterization of different patterns of inflammation in RuV-associated granulomas may lead to a better understanding of the role of RuV in chronic tissues inflammation and associated pathologies and identify new targets for therapeutic interventions.

## Materials and Methods

### Ethics Statement

Diagnostic samples were obtained from all patients as a part of clinical care with provision of informed consent by attending physicians. Samples were submitted to the Rubella Laboratory (Centers for Disease Control and Prevention (CDC), Atlanta, USA) for molecular testing, virus culture, and rubella serology. Testing was performed as a part of the reference and surveillance responsibilities of the CDC laboratory. Since RuV analyses were conducted for the purpose of public health response, this work was determined not to be research in humans by the CDC Institutional Review Board (IRB). Types of specimens collected for diagnostic purposes are indicated in **Table 1** for each patient. Archived formalin-fixed paraffin-embedded (FFPE) specimens were tested by the CDC laboratory with nondisclosure of patient information, which was determined to be ethically acceptable by the CDC IRB. This work was determined to be non-applicable for human subject regulations (project determination numbers P_2017_DVD_Icenogle_415 and P_2017_DVD_Icenogle_330).

**Table 1:** Rubella virus testing of clinical samples from 28 IEI patients.

Pt #	Age (years) at Sampling	Tissue	IHC Score^a^	Granuloma Pattern^b^	Real-Time RT-PCR (RuV Genotype)^c^	Virus Isolation^d^	Rubella Serology
1	15	FFPE skin, finger, old lesion	P 1+	M-type			
	16	FFPE liver	P 4+	M-type			
	16	FFPE skin, finger, new lesion	P 1+	M-type			
	17	serum			Neg	Neg	IgM-, IgG+, NT_50_ = 9200^e^
	17	skin biopsy, R index finger^f^			P (iVDRV)	P	
	17	skin biopsy, R index finger, after 6 wks NTZ^g^			P (iVDRV)	P	
	18	skin biopsy, R index finger, after 3.5 months NTZ			P (iVDRV)	P	
	18	lymph node, autopsy			Neg	Neg	
	18	tumor, arm, autopsy			Neg	Neg	
	18	elbow synovial tissue, autopsy			Neg	Neg	
	18	wrist synovial tissue, autopsy			Neg	Neg	
	18	normal skin, arm, autopsy			Neg	Neg	
	18	nerve tissue, autopsy			Neg	Neg	
	18	elbow joint fluid, autopsy			Neg		
	18	wrist joint fluid, autopsy			P^h^		
	18	bone marrow aspirate, autopsy			Neg		
2	5	FFPE skin 1	P 3+	M(n) -type			
	5	FFPE skin 2	P 2+	M(n) -type			
	5	NP swab			Neg	Neg	
3	59	FFPE skin 1	P 1+	N-type			
	59	FFPE skin 2	Neg				
4	3	FFPE skin	P 1+	M-type			
	3	FFPE bone marrow core	P 1+	M-type			
5	11	FFPE skin	P 2+	M-type			
	12	serum					IgM-, IgG+; NT_50_ = 6400
	12	NP swab			P (iVDRV)	Neg	
6	2	FFPE GI biopsy	Neg				
	2	FFPE bone marrow clot	P 1+				
7	7	FFPE skin	P 3+	N-type			
8	74	FFPE skin	P 3+	N-type			
9	14	FFPE bone marrow core	1+				
	14	FFPE liver	Neg				
	14	FFPE lung	P 1+	DNI-type			
10	6	FFPE left axillary lymph node	P 1+				
11	13	FFPE groin tissue^i^	P 4+	N-type			
12	24	FFPE bone marrow clot	P 4+				
	24	FFPE bone marrow core	P 3+				
	24	FFPE brain	P 1+	DNI-type			
13	20	FFPE liver	P 2+	M-type			
	20	serum					IgM+, IgG+
14	27	FFPE skin	P 3+	M-type			
15	37	FFPE skin	P 3+	M-type			
16	12	FFPE skin	P 4+	M-type			
	12	FFPE bone marrow core	Neg				
	12	FFPE bone marrow clot	P1+				
	12	FFPE GI biopsy 1	P 4+	DNI-type			
	12	FFPE GI biopsy 2	P 2+	DNI-type			
	12	whole blood			Neg		IgM-, IgG+
	12	NP swab			P (iVDRV)	Neg	
17	4	skin biopsy	P1+	M-type	Neg	Neg	
	4	FFPE skin	P 1+	M-type			
18	1.6	FFPE skin	P 4+	M(n) -type			
	1.6	skin biopsy			P (iVDRV)		
	1.6	serum					IgM+ (grey zone), IgG+
19	32	FFPE skin, leg right lateral	P 2+	M-type			
	32	FFPE skin, arm, left upper	P 2+	M-type			
	33	FFPE skin, arm, left upper	P 4+	M-type			
	37	FFPE skin, arm, left upper	P 1+	M-type			
	38	NP swab			Neg	Neg	
	38	urine			Neg		
	38	skin biopsy			Neg	Neg	
	38	serum			Neg		IgM-, IgG+; NT=1280
20	11	FFPE skin	P 4+	N-type			
	11	FFPE bone marrow core	Neg				
	11	NP swab			P (iVDRV)	Neg	
	11	whole blood					IgM+, IgG+; NT=400
21	2	FFPE skin, left thigh	P 4+	N-type			
	2	FFPE bone marrow core	P 4+				
22	11	FFPE skin, R index finger	P 3+	M-type	P (iVDRV)		
23	4	FFPE skin 1	P 4+	M(n)-type			
	4	FFPE skin 2	P 3+	M(n)-type			
24	1.4	FFPE skin 1	P 4+	M-type	Neg		
	1.4	FFPE skin 2	P 4+	M-type	Neg		
	5	FFPE brain, autopsy	1+				
	5	FFPE myocardium, autopsy	Neg				
	5	FFPE kidney, autopsy	Neg				
	5	FFPE adrenal, autopsy	Neg				
	5	FFPE ovary, autopsy	Neg				
	5	FFPE lymph node, autopsy	Neg				
	5	FFPE colon, autopsy	Neg				
	5	FFPE lung, autopsy	P 2+	DNI-type			
	5	FFPE skeletal muscle, autopsy	Neg				
	5	FFPE pancreas, autopsy	P 1+	DNI-type			
	5	FFPE hippocampus, autopsy	Neg				
25	12	FFPE skin, hand	P3+	M(n) -type			
26	19	FFPE spleen	P1+	DNI-type			
27	3.8	FFPE brain	P3+	DNI-type			
28		FFPE skin	P4+	M(n) -type			
		serum					IgM-, IgG+
		skin biopsy			P^h^		

a Positive (P) IHC staining for RuV capsid was scored on a scale from 1+ to 4+ based on the intensity of staining and a number of positively stained cells. Neg – negative.

b Granuloma pattens based on double IHC staining for RVC and cell type markers.

c RuV RT-PCR was resulted either positive (P) or negative (Neg). RuV genotype was determined by sequencing and indicated in parentheses.

d Virus isolation was done using Vero cells, P -positive, Neg- negative.

e NT_50_– RuV neutralization titer.

f Virus isolation and sequencing were previously reported for this biopsy sample collected prior to NTZ treatment (20).

g NTZ – nitazoxanide treatment. A manuscript is currently in preparation to describe RuV RNA quantitation in the pre- and post-treatment biopsies, the comparison of the genomic sequences and quasispecies composition of the viruses isolated pre- and post-treatments, and sensitivities of the recovered viruses to NTZ.

h Not sufficient material for sequencing.

i This tissue was collected during the failed attempt to collect biopsy of the groin lymph node.

### Patient Survey of Clinical and Immunological Characteristics

Patients with granulomas of unknown etiology were identified through a network of colleagues and personal communications. A 22-question survey was designed by Kathleen E. Sullivan and administered to 24 physicians attending immunodeficient patients with granulomas to assess the following: patient current age and age of IEI manifestation and diagnosis, IEI genetic cause, mean CD3 and naïve CD3 counts, chronic infections and autoimmunity, age at MMR, age at granuloma onset, granuloma locations at onset, organs involved in inflammation, age at granuloma biopsy and description of biopsy pathologies, granuloma treatments and outcomes. The survey was completed between January 2021 and March 2021.

### Fluorescent Immunohistochemical Staining (IHC) and Imaging

The presence of rubella antigen in FFPE tissue sections was detected by fluorescent IHC using mouse monoclonal antibody against rubella virus capsid (RVC) (Abcam, cat# ab34749) as previously described in detail (16). RuV-infected cell types were determined by double immunostaining with either rabbit monoclonal antibodies against CD3 (Abcam, cat# ab16669), CD14 (Abcam, cat# ab133335), CD20 (Abcam, cat# ab78237), CD34 (Abcam, cat# ab254022), CD68 (Cell Signaling, cat# 76437), CD163 (Abcam, cat# ab 182422), CD71 (Abcam, cat# ab108985), myeloperoxidase (MPO) (Abcam, cat# ab208670), or rabbit polyclonal antibody against CD206 (Abcam, cat# ab64693), and von Willebrand Factor (vWF) (Sigma, cat# F3520). FFPE tissue sections of normal human brain, lymph node, pancreas, spleen, lung, skin, colon, kidney, and liver (Newcomer Supply, Middleton, WI) were used as normal tissue controls. Mouse monoclonal antibodies against measles nucleoprotein (Millipore, cat# MAB8906), mumps nucleoprotein (Abcam, cat#ab9880), or mouse monoclonal antibody cocktail against varicella zoster virus were used as negative controls for granuloma staining. IHC stain was visually scored as follows based on the number of RVC^+^ cells and intensity of RVC immunostaining: 1=weak, 2=moderate, 3=strong, 4=very strong. Images were acquired using either a Zeiss epifluorescence microscope AxioImager.A1 or a Zeiss laser scanning confocal microscope LSM-800 (Carl Zeiss Microscopy, LLC). To image large tissue sections, multiple overlapping fields were acquired and stitched using a ZenBlue v3.3 software (Carl Zeiss Microscopy, LLC).

### RuV Molecular Analysis

Viral RNA isolation, RuV RT-PCR, viral RNA sequencing and phylogenetic analysis have been described previously (20).

### Rubella Serology

Rubella IgM and IgG testing was performed with a Rubella IgM Capture EIA Kit (Diamedix, Miami, FL) and ZEUS ELISA Rubella IgG Test System (ZEUS Scientific, Branchburg, NJ). RuV neutralization assay was performed as previously described (20). The neutralization titer (NT_50_) of a serum sample was expressed as the reciprocal of the dilution of the serum that neutralized 50% of added RA27/3 vaccine virus strain.

## Results

### Characteristics of the Patient Cohort

We identified 28 patients with inborn and acquired errors of immunity with RuV^+^ granulomatous inflammation in one or more organs. **Table S1** summarizes clinical, immunological, and genetic characteristics of the patients. Six patients (21%) are deceased due to various co-morbidities.

### Underlying Inborn and Acquired Errors of Immunity

**Figure 1:**
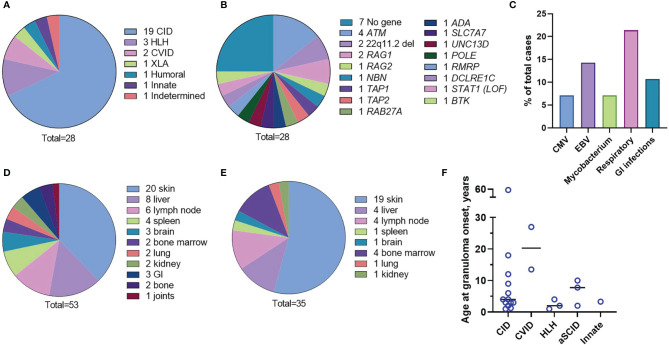
Characteristics of the patient cohort. Underlying immunodeficiencies **(A)**. Genetic causes **(B)**. Frequency of chronic infections **(C)**. Organs involved in inflammation **(D)**. Granuloma location at onset **(E)**. Age of the patients at the granuloma onset by the IEI type **(F)**.

The underlying immunodeficiencies were categorized according to the International Union of Immunologic Societies (IUIS) classification (1, 2) (**Table S1** and **Figure 1A**). The most frequent phenotype, combined immunodeficiency (CID), was found in 19 (67.8%) cases, and all were characterized by reduced CD3 T-cell counts, 16 had a genetic diagnosis, and 8 had phenotyping of naïve/memory T cells available showing loss of naivety. Two CID patients (P3 and P23) were without a genetic diagnosis and without naïve T-cell counts but had T-cell lymphopenia and opportunistic infections. Within this group, there were four patients with atypical severe combined immunodeficiency (aSCID) and two patients with DiGeorge syndrome (22q11 deletion syndrome). Additionally, there were two patients with CVID, one patient with X-linked agammaglobulinemia (XLA), three patients with hemophagocytic lymphohistiocytosis (HLH), one patient with a defect of innate immunity (i.e., biallelic STAT1 loss-of-function), one patient with a secondary immunodeficiency (lymphopenia), most likely due to severe malnutrition, and one patient with an IgG_2_ and IgA deficiency without a genetic explanation. The median age of immunodeficiency manifestation was 2 years (age range 0-59 years). The genetic cause of IEI was determined for 21 (75%) cases at median age of 5 years (age range 0-37 years). The syndromic CID ataxia telangiectasia with mutations in the *ATM* gene was the most common single etiology found in four patients (**Table S1** and **Figure 1B**).

### Clinical and Immunologic Phenotypes

Respiratory tract infections were the most frequently observed infections; the most common infectious agents identified were Epstein-Barr Virus (EBV), cytomegalovirus (CMV), and mycobacteria (**Figure 1C** and **Table S1**). Different organs were involved in granulomatous disease manifestations with skin, liver, and lymph node being the most frequent (**Figure 1D** and **Table S1**). Granulomas were initially identified in the inflamed tissues after H&E staining of the tissue biopsies. There were multiple locations of granulomas at disease onset; cutaneous granulomas were the most frequent first presentation (**Figure 1E** and **Table S1**). Patient 4 (P4), P8, and P27 had granulomas located in multiple organs at disease onset; bone marrow granulomas were found in all these three cases (**Table S1**). The overall median age at granuloma onset was four years (age range 1 to 59 years) and appeared to depend on the IEI type. For instance, patients with HLH (P5, P17, P18) developed granulomas soon after vaccination while patients with CVID (P9, P19) developed granulomas later in life (**Figure 1F** and **Table S1**). The course of the disease was complicated by end organ damage in several patients (**Table S1**). Low CD3^+^ T-cell counts (<1,600 cells/µl) were detected in 23/28 (82.1%) patients.

### MMR Vaccination

The first dose of MMR vaccine was given to 22 (78.6%) patients at a median age of 1 year (age range 0.8 – 2.5 years). P3 was vaccinated but the vaccination date is unknown. P7 was not vaccinated but community transmission of wild type RuV was documented. The vaccination status of four patients (P12, P14, P15, P19) is unknown. The second MMR dose was given to 13 (46.4%) patients at a median age of 2.5 years (age range 1.25 – 5.25 years). Except P26, 21/22 (95.5%) individuals were vaccinated prior to an IEI diagnosis.

### Detection and Sequencing of RuV RNA

Fresh frozen skin samples were collected from five (17.9%) patients. P1, P18, P28 biopsies (60%) were tested positive for the presence of RuV RNA by RT-PCR (**Table 1**). RuV was not detected in the unfixed skin biopsies of P17 and P19 by RT-PCR even though the other skin biopsies of these patients were positive by IHC; this could reflect a sampling problem as RuV is not distributed evenly in the lesions. Multiple tissue samples of P1 were tested. All three skin biopsies collected prior and after nitazoxanide therapy were positive by RT-PCR and cell culture, thus confirming the failure to clear RuV cutaneous infection by this treatment. One sample (wrist joint fluid) out of nine collected after upper limb amputation of P1 was also RuV^+^ by RT-PCR. Nasopharyngeal swabs were available for five (17.9%) patients, and three of these (60%) were RuV^+^ by RT-PCR but no live virus was recovered. We were able to amplify the RuV genotyping fragment from RNA isolated from P22 FFPE skin tissue in one out of three (33%) available for analysis. Sequencing confirmed the presence of iVDRVs in six out of seven (85.7%) PCR-positive cases. A genotype of RuV (wild type or iVDRV) in lesions of other patients was not determined.

### RuV Serological Findings

Rubella serology was performed for eight (28.6%) patients (**Table 1**). Three (37.5%) patients (P1, P13, P20) had persistent RuV IgM antibody. All four patients, for which RuV neutralization assay was done, had NT_50_ titers of RuV neutralizing antibody ranging from 400 to 9,200, which were substantially higher than typically found in immunologically normal vaccinated individuals (NT_50_<100). These data were consistent with previously reported findings (20) and substantiate the proposition that high levels of RuV neutralizing antibodies can potentially serve as a marker of persistent rubella infection in individuals with IEI.

### Therapeutic Efforts and Outcomes

Various therapies were implemented (**Table 2**). Supportive treatments, such as antimicrobials, antifungals, and IgG supplementation, did not result in relevant improvement. Steroids and disease modifying antirheumatic drugs (DMARDs) provided some resolution of inflammation in several cases. Treatments with biologicals, such as TNF-α and IL-1R antagonists led to reduction of lesions in 3/5 (60%) patients. Curative treatment for immunodeficiency (hematopoietic cell transplantation) resulted in resolution of the lesions in those patients (5 of 7) who survived the procedure.

**Table 2:** Treatment response in 28 patients with granulomas.

Treatments	Patient #	Outcomes
Antibiotics	2, 6, 16, 18	no effect
Antifungals, topical or oral	2, 4, 6, 16,17	no effect
Steroids, oral	8, 9, 10, 12, 16,17, 20, 24	1/8 improvement (P8)
Steroids, topical	2, 3, 4, 8, 18, 21, 24, 28	2/8 improvement (P3, P18)
Steroids, intralesional	8	no effect
IVIG	10, 16, 17, 18, 21, 24, 28	no effect
DMARDs (mycophenolate mofetil, hydroxychloroquine, methotrexate)	3, 9, 17,19, 27	1/5 improvement (P19)
IL-1R antagonist (anakinra)	7, 27	1/2 improvement (P7)
TNF-α antagonist (etanercept, adalimumab, infliximab)	1, 4, 19	2/3 improvement (P4, P19)
Anti-CD20 (rituximab)	9, 10, 24, 27	no effect
IL-1 beta antagonist (canakinumab)	17	no effect
CD80/CD86 antagonist (abatacept)	23	no effect
Nitazoxanide	1, 18	1/2 fewer new lesions (P18)
Hematopoietic cell transplantation	7, 12, 16, 18, 20, 24, 28	5/7 granulomas resolved, P20 and P24 did not survive the procedure

IVIG, intravenous immunoglobulins; DMARDs, disease modifying antirheumatic drugs.

### Granuloma Characteristics by Immunofluorescent Immunohistochemistry

A total of 60 FFPE tissue blocks from 28 patients were available for analysis. The presence of RuV capsid protein (RVC) in 27 skin samples and 33 other tissues was detected by fluorescent immunohistochemistry (IHC) (**Table 1**). To determine the cell composition and spatial organization of the granulomas, all slides were double stained for RVC and either for CD206 (M2 macrophage marker) or MPO (cytoplasmic neutrophil marker). Selected slides were also double stained for RVC and CD68 (pan-macrophage), CD163 (a marker for M2 macrophage subset, which only partially overlaps with CD206^+^ M2 macrophage subset), CD3 (T cells), CD14 (monocytes), CD20 (B cells), CD34 (stem cells), CD71 (erythroid precursors), vWF (endothelial cells). As a negative control, the slides were stained with antibodies to other live-attenuated vaccine viruses, i.e., measles, mumps, or varicella zoster virus. Granulomas on all slides were negative for these control viral antigens (not shown).

### Cutaneous Granulomas

RVC^+^ granulomas were detected in skin biopsies of all 20 patients who had cutaneous lesions. Multiple skin biopsies were available for six patients; 5/6 patients had all sites positive for RVC, and one patient (P3) had a single RVC^+^ biopsy site out of two sites tested. Skin biopsies of 15/20 (75%) patients contained well-formed epithelioid granulomas. These granulomas were either non-necrotizing (10/15) or necrotizing (5/15).

#### M-Type RuV-Associated Granuloma

**Figure 2:**
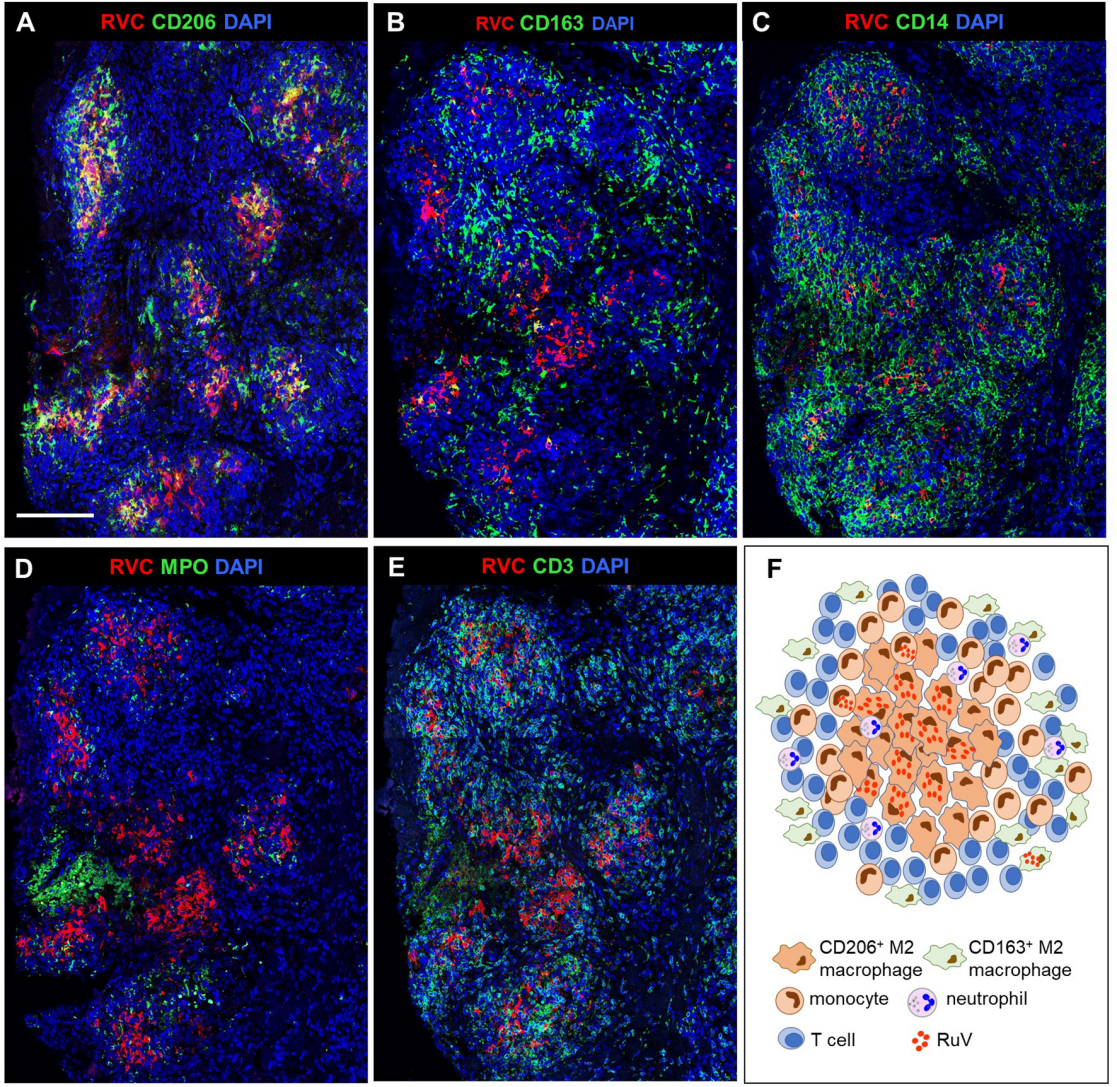
A structure of M-type RuV-associated cutaneous granuloma and its cellular elements. Histological double immunofluorescent staining of sequential tissue sections (P18) for RVC (red) and one of the cell type markers (green) shows the presence of RVC predominantly in CD206^+^ M2 macrophages **(A)** and infrequently in CD163^+^ M2 macrophages **(B)**, CD14+ monocytes **(C)** and in sporadic MPO+ neutrophils **(D)**. Layers of many RVC^-^ CD3^+^ T cells surround RVC^+^/CD206^+^ granuloma centers **(E)**. Scale bar: 200 µm. Schematic of M-type RuV-associated granuloma pattern **(F)**.

**Figure 3:**
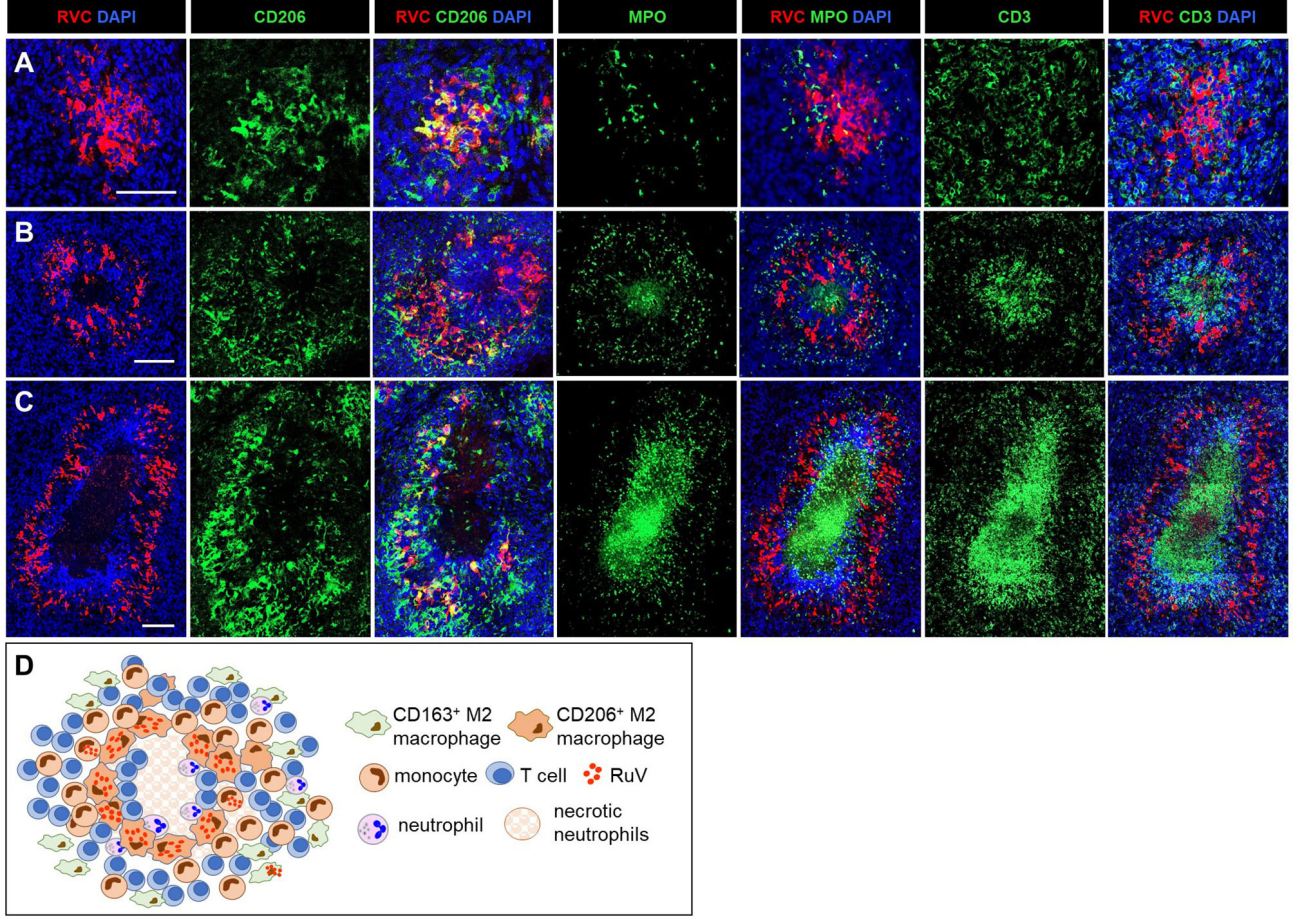
A progression of cutaneous granuloma from M-type to M(n)-type. Histological double staining of sequential tissue slides (P18) for either RVC (red) and one of the cell type markers (green). Non-necrotizing M-type granuloma contains RVC^+^CD206^+^ M2 macrophages in the center with infrequent, mainly RVC^-^ neutrophils and abundant surrounding RVC^-^CD3^+^ T cells **(A)**. Necrotizing M(n)-type granulomas with MPO^+^ and CD3 ^+^ staining of the necrotic centers surrounded by RVC^+^CD206^+^ macrophages **(B, C)**. Notice a rim CD3^+^ T cells located between acellular necrotic center and RVC^+^CD206^+^ macrophages **(C)**. Scale bar: 100 µM. Schematic of M(n)-type RuV-associated granuloma pattern **(D)**.

We use as the designation for non-necrotizing granulomas with a clearly defined central core of interconnected RVC^+^CD206^+^ M2 macrophages with epithelioid morphology (**Figures 2A, F**, **3A**). RVC antigen was diffusely distributed in the cytoplasm of these macrophages. RVC^+^CD206^+^ Langhans giant cells were present in the granuloma cores of five patients (P3, P14, P15, P16, and P19) (**Figure S1**). A CD163^+^ M2 macrophage subset with a small number of RVC^+^ cells was peripherally located (**Figure 2B**). Some RVC^+^ M2 macrophages in granuloma cores were CD206^high^CD163^low^ double positive. Abundant CD14^+^ monocytes were also present in M-type granulomas, and monocytes adjacent to RVC^+^CD206^+^ foci were often positive for RVC (**Figure 2C**). Variable but usually small numbers of MPO^+^ neutrophils were randomly distributed in the M-type granuloma centers among the RVC^+^ macrophages; most of them were negative for RVC (**Figure 2D**, 3A). None of the numerous CD3^+^ T cells stained positive for RVC. T cells were randomly distributed throughout the granulomas; some of them were in close contact with RVC^+^ macrophages (**Figures 2E**, **3A**). Only a small number of CD20^+^ B cells were localized at the periphery of all granuloma types (not shown).

#### M(n)-Type RuV-Associated Granuloma

We use as the designation for necrotizing epithelioid granulomas consisting of a ring of RVC^+^CD206^+^ M2 macrophages surrounding acellular necrotic centers (**Figures 3B–D**). M(n)-type granulomas often contained a mixture of necrotizing and non-necrotizing granulomas in a single biopsy each likely being different stages of granuloma development. Densely packed CD3^+^ T cells and some neutrophils were present between the ring of RVC^+^CD206^+^ macrophages and necrotic centers. The necrotic centers contained cell debris that were strongly stained for MPO and CD3 (**Figures 3B, C**). Weak punctuate staining for RVC and CD206 was also detected in the necrotic centers (**Figure 3C**). These data suggest that T cells and neutrophils migrated into granulomas, participated in the destruction of RuV infected macrophages and died by necrosis thus forming necrotic centers of M(n)-type granulomas. Monocytes and CD163^+^ M2 macrophage were abundant and their distribution in M(n)-type granulomas was like those in M-type granulomas.

#### N-Type RuV-Associated Granuloma

**Figure 4:**
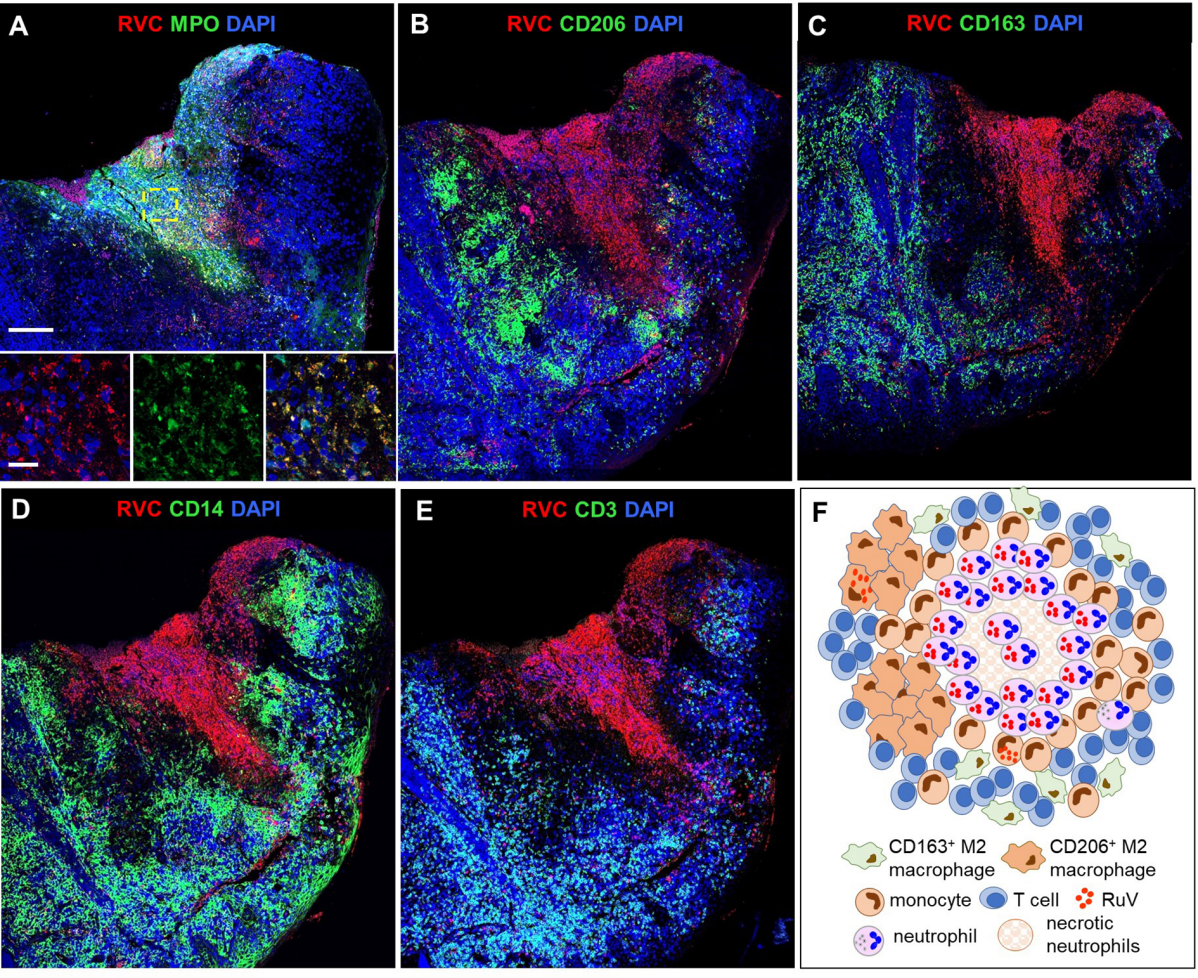
A structure of N-type RuV-associated cutaneous granuloma and its cellular elements. Histological double immunofluorescent staining of sequential tissue sections of skin biopsy (P21) for RVC (red) and one of the cell type markers (green) shows predominant RVC staining of MPO^+^ neutrophils **(A)**, infrequent RVC staining of CD206^+^ M2 macrophages **(B)**, CD163^+^ M2 macrophages **(C)**, and CD14^+^ monocytes **(D)**. Numerous RVC^-^CD3^+^ T cells surround the RVC^+^ neutrophil core **(E)**. Scale bars: 200 µm and 20 µm (inlet). Schematic of N-type RuV-associated granuloma pattern **(F)**.

We use as the designation for necrotizing neutrophilic granulomas with neutrophils being the main cell type harboring RuV antigen (**Figure 4**). The skin biopsies of 5/20 (25%) patients (P3, P7, P8, P20, P21) contained N-type granulomas. The cores of these granulomas consisted of many tightly packed MPO^+^RVC^+^ neutrophils and were devoid of other cell types (**Figure 4A**). There were also multiple areas of necrosis in the core. Clusters of CD206^+^ M2 macrophages with infrequent RVC^+^ cells were found adjacent to the N-type cores (**Figure 4B**). Monocytes, CD163^+^ M2 macrophages and T cells were abundant and distributed around the granuloma core (**Figures 4C–F**).

**Figure 5:**
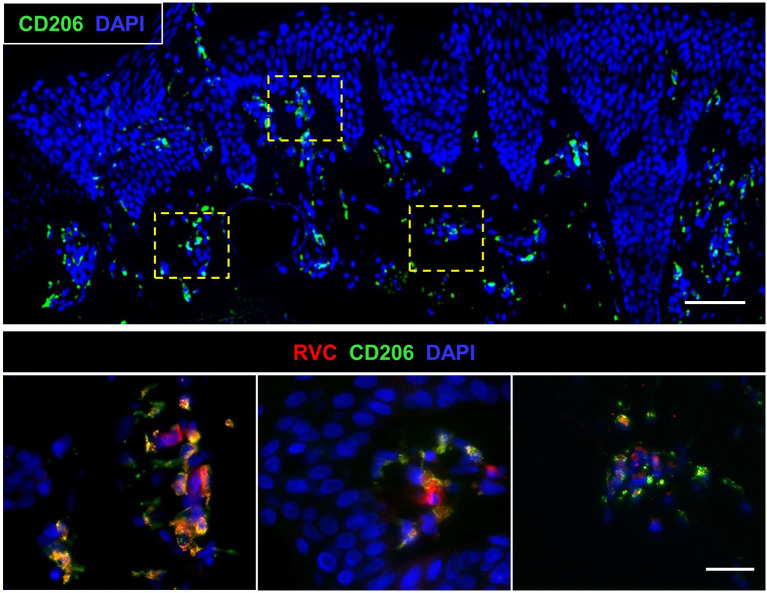
Biopsy of a newly developed cutaneous lesion (P1). Double histological immunofluorescent staining shows small clusters of RVC^+^CD206^+^ M2 macrophages under the epidermis. Scale bars: 100 µm (top panel) and 20 µm (bottom panel).

Visually determined RVC IHC positivity scores varied among different biopsies of the same patient as well as among biopsies of different patients. The IHC scores and granuloma types are listed in **Table 1**. Notably, the granuloma patterns in individuals with multiple biopsies did not change over time: multiple cutaneous biopsies showed the same granuloma patterns, M-types in P1 and P19 or M(n)-types in P2 and P23. No correlation was detected between the patterns of RuV-associated granulomas and age of granuloma onset, age at granuloma sampling, and the time elapsed between granuloma onset and sampling (**Figure S2**) nor the IEI type or T-cell counts (**Table S1**),

IHC analysis of a newly developed skin lesion of P1 did not reveal a clearly defined granuloma (**Figure 5**). Instead, there was an accumulation of several small clusters of RVC^+^ M2 macrophages (10-50 cells each), which were localized under the epidermis. Each of these small macrophage clusters may represent an early stage of granuloma formation. The skin biopsy of an older lesion (not shown) and the liver biopsy (**Figure S3**) of P1 contained typical M-type granulomas. This observation suggests that RuV-infected macrophages could be a vehicle of lateral dissemination of granulomas in the affected skin as suggested for *Mycobacteria-*induced granulomas in tuberculosis (26).

### Granulomas in Extracutaneous Sites

#### Liver

The liver biopsies from 2/3 patients (P1, P13) had M-type RVC^+^ granulomas comparable with cutaneous M-type RVC^+^ granulomas. RVC antigen was detected in CD206^+^ macrophages and multinucleated giant Langhans cells in the granuloma centers and infrequent RVC^+^MPO^+^ neutrophils were found in the surrounding areas (**Figure S3**).

#### Gastrointestinal (GI) Tract

**Figure 6:**
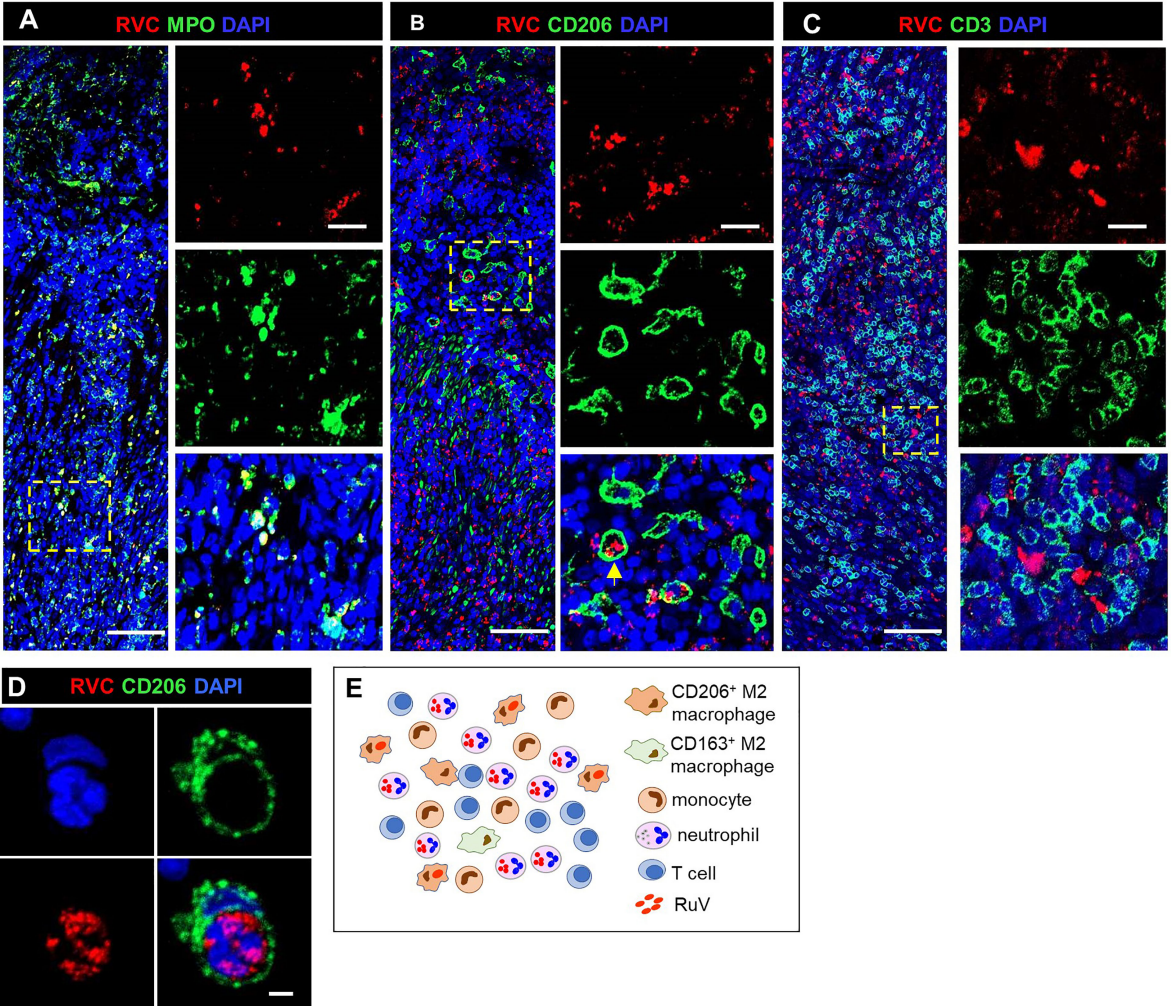
A structure of DNI-type RuV-associated granuloma and its cellular elements. Histological double immunofluorescent staining for RVC and either MPO **(A)**, CD206 **(B, D)**, or CD3 **(C)** showing RVC^+^MPO^+^ neutrophils, RVC^+^CD206^+^ macrophages with globular RVC likely in a phagosome (yellow arrow) and abundant RVC^-^CD3^+^ T cells in the inflamed GI tissue of P16 **(A–C)**. RVC^+^ neutrophil phagocytized by CD206^+^ macrophage **(D)**. Scale bars: 100 µm **(A-C)**, 20 µm (inlets), and 2 µm **(D)**. Schematic of DNI-type RuV- associated granuloma pattern **(E)**.

GI biopsies were available for two patients: P2 had one RVC^-^ GI biopsy and P16 had two RVC^+^ GI biopsies. P16, who presented with HLH, had sustained small jejunal perforations; disseminated CMV and *Histoplasma* spp. infections were identified in addition to RuV (**Table S1**). Predominant RVC^+^ cells were neutrophils (**Figure 6A**) and well-formed granulomas with clearly defined macrophage or neutrophil cores as present in skin biopsies were not observed. We defined *DNI-type (diffuse neutrophil inflammation) of RuV-associated granuloma* as a disorganized aggregation of abundant RVC^+^ neutrophils intermixed with macrophages (some RVC^+^) and RVC^-^ T cells (**Figures 6B–D**). CD206^+^RVC^+^ cells in DNI-type granulomas were morphologically distinct from those in the centers of M-type granulomas: rounded with shorter cell processes, not interconnected, with CD206 protein localized mainly in the plasma membrane and RVC antigen localized in large globular structures that most likely were phagosomes (**Figure 6B**). RVC^+^ neutrophils phagocytized by CD206^+^ macrophages were also observed (**Figure 6D**).

#### Brain

**Figure 7:**
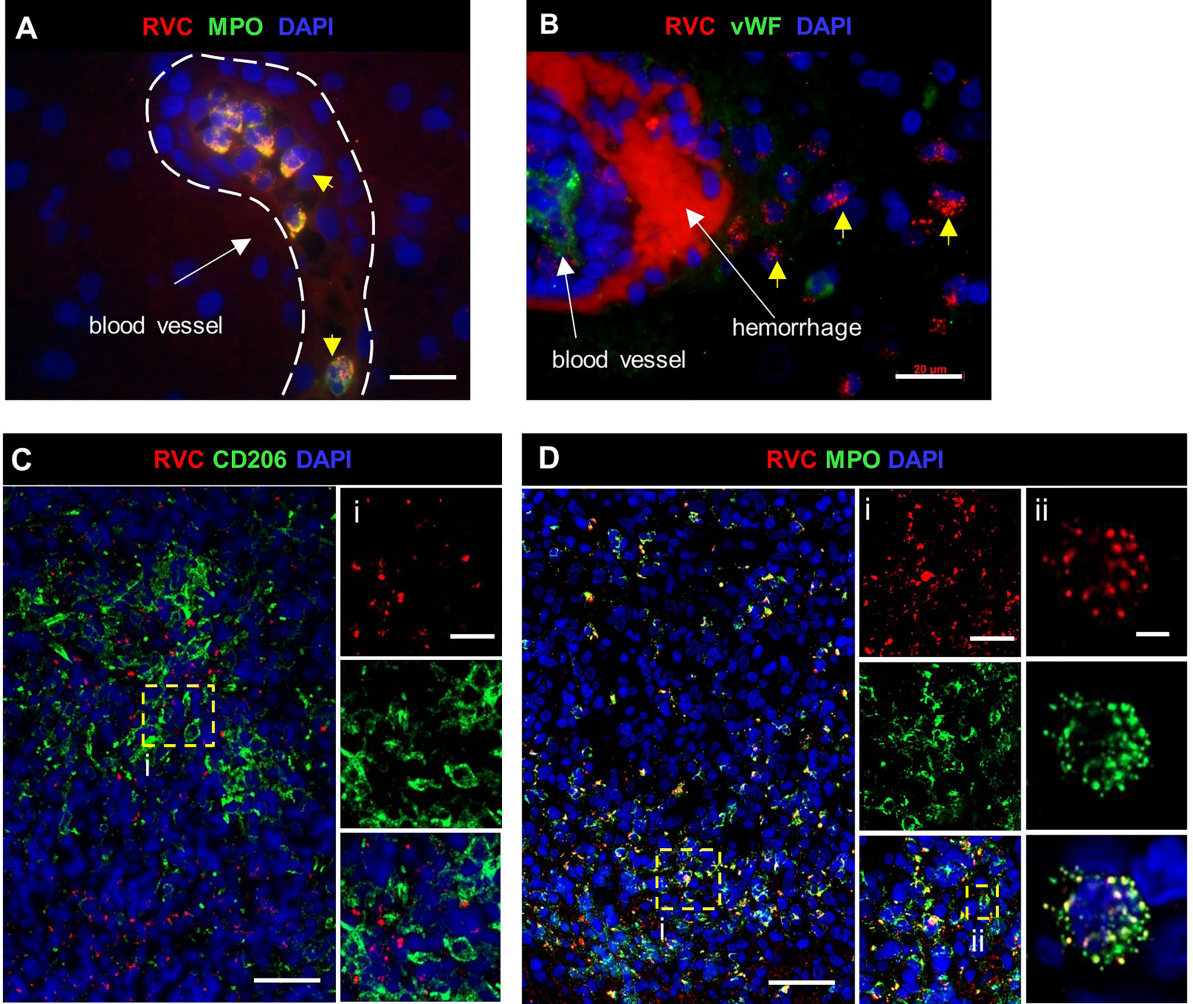
RuV in brain neutrophils. Histological double immunofluorescent staining for RVC **(A–D)** and either MPO **(A, D)**, vWF **(B)** or CD206 **(C)**, showing RVC^+^ neutrophils in the lumen of blood vessels, perivascular cuff, and around the area of hemorrhage (yellow arrows) in P12 brain **(A, B)**. Numerous RVC^+^ neutrophils surrounding RVC^-^CD206^+^ granuloma in P27 brain **(C, D)**. Notice a colocalization of MPO granules with RVC **(Dii)**. Scale bars: 20 µm **(A, B, Ci, Di)**. 50 µm **(C, D)**, and 2 µm **(Dii)**.

In brain biopsies, MPO^+^RVC^+^ neutrophils were found inside capillaries, in perivascular regions, and near parenchymal hemorrhage (P12, P24, P27) (**Figures 7A, B**). In addition, brain biopsies of P12 and P27 with clinical encephalitis (**Table S1**) contained several small granulomas with RVC^-^CD206^+^ M2 macrophage cores and RVC^+^ neutrophils intermixed with other immune cells surrounding RVC^-^ granulomas and throughout the brain parenchyma (DNI-type granuloma). RVC^+^ neutrophil infiltration was mild in the brain from P12, but severe, with multiple arears of necrosis, in the brain of P27 (**Figures 7C, D**). The pathology report for P12, with XLA and large granular lymphocyte (LGL) disease on treatment with cyclophosphamide, described perivascular cuffs of lymphocytes, neurophagia, and glial nodules characteristic of viral infection. P27 with biallelic STAT1 loss-of-function had glial fibrillary acidic protein (GFAP) autoimmune encephalitis. These data suggest that, while RuV infection may not be the initial trigger of inflammation in the brain, the influx of RVC^+^ neutrophils can bring additional antigen to the site and exacerbate the inflammation.

#### Spleen

Numerous granulomas with RVC^-^CD206^+^ M2 macrophage containing centers in the P26 spleen sample were surrounded by RVC^+^ neutrophils (DNI-type granuloma). Occasional RVC^+^CD206^+^ M2 macrophages were also observed (**Figure S4**).

#### Lung, Pancreas

RVC^+^ neutrophils were found randomly distributed in parenchyma of the P9 lung biopsy and the P24 pancreas and lung (DNI-type granuloma) (**Figure S5**). In other P24 autopsy tissues, RVC^+^ neutrophils were detected only in lumens of blood vessels.

#### Lymph Node

One of two available lymph node samples (P10) were RuV positive. RVC^+^ cells were CD14^+^ monocytes and sporadic CD68^+^ macrophages in the paracortex area (**Figure S6**). Granuloma type was not defined for this sample.

#### Bone Marrow

**Figure 8:**
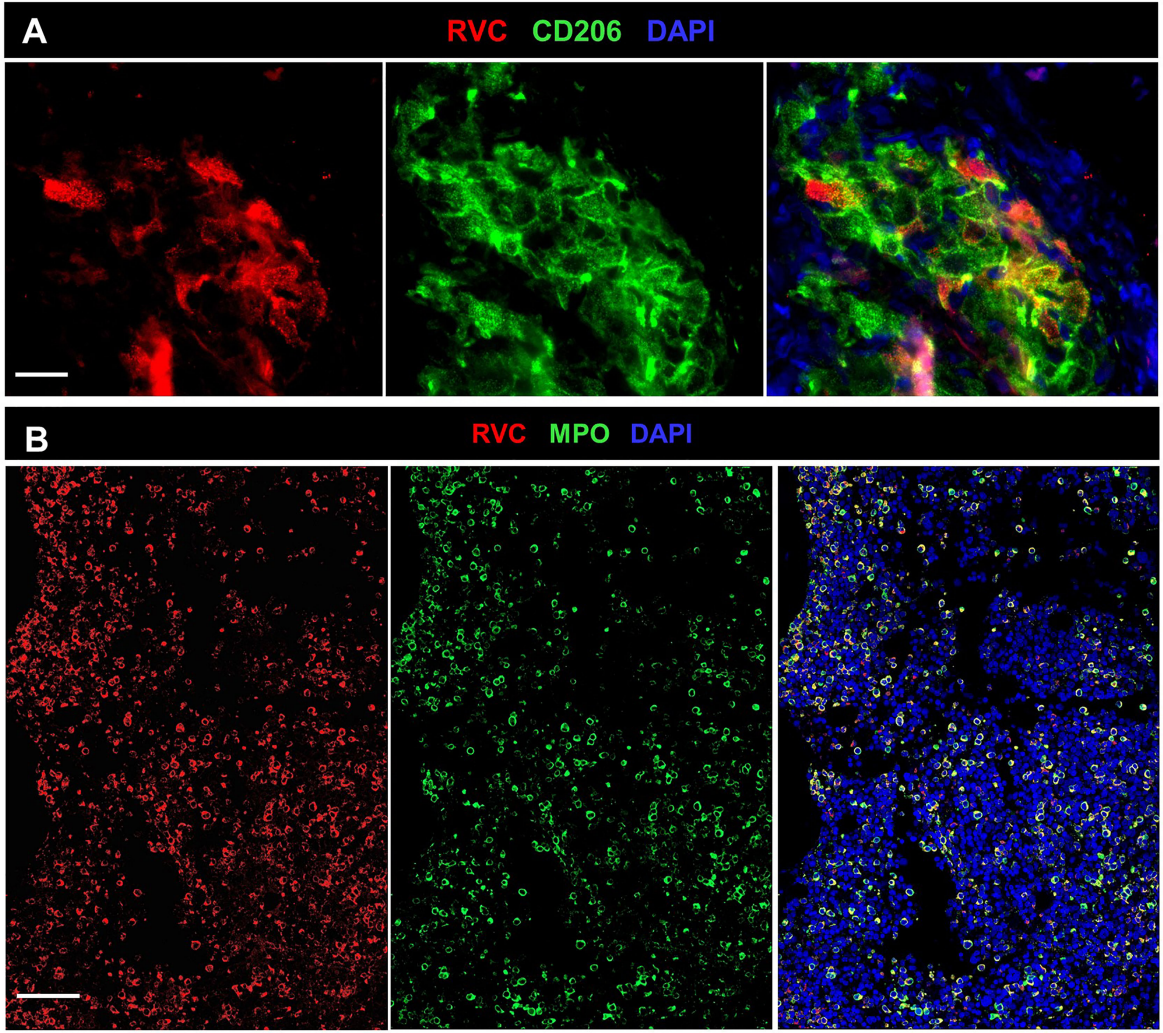
RuV in bone marrow. Histological immunofluorescent staining of bone marrow core biopsies of P4 **(A)** and P21 **(B)** for RVC **(A, B)** and either for CD206 **(A)** or MPO **(B)** shows the presence of RuV in M2 macrophages **(A)** and neutrophils **(B)**. Scale bars: 20 µm **(A)** and 100 µm **(B)**.

Granulomas were detected in bone marrow samples of P4, P6, P8, and P27 by H&E staining (**Table S1**). P4 core biopsy, the only one available for RVC IHC staining, contained M-type RuV-associated granulomas and a small number of RVC^+^MPO^+^ neutrophils (**Figure 8A**). Pathological changes, such as hypocellularity, focal fibrosis, and lymphoid aggregates were found in one bone marrow sample (P12); almost all neutrophils in this sample were RVC^+^. No granulomas or sign of inflammation were noted in bone marrow samples of P9, P16, P20, and P21 (**Table S1**). Nonetheless, P9 core biopsy contained sporadic RVC^+^MPO^+^ neutrophils and almost all neutrophils in P21 bone marrow were RVC^+^ (**Figure 8B**). No RVC^+^ cells were detected in P16 and P20 core biopsies, but the presence of sporadic RVC^+^ neutrophils in P16 bone marrow clot suggested either poor sampling or, more likely, uneven distribution of infected neutrophils in bone marrow. Infrequent RVC^+^CD68^+^ macrophages and RVC^+^CD14^+^ monocytes were also detected in bone marrow samples, while CD34^+^ stem cells and CD71^+^ erythroid precursors were RVC-negative (not shown). The presence of RVC^+^ neutrophils in otherwise normal bone marrow samples suggest that bone marrow is not a site of RuV-associated inflammation but rather a persistence niche for RuV.

## Discussion

Herein, we report the first study of spatial and cellular organization of RuV-associated granulomas in subjects with inborn and acquired errors of immunity using fluorescent immunohistochemical staining of lesion biopsies. One of the key findings is that, in addition to macrophages, neutrophils in granulomas contained the RVC antigen. The large number of granuloma cases analyzed in detail in this manuscript allowed identification of four distinct granuloma patterns, which were classified based on the identity of cells harboring RVC antigen and their distribution in the lesions. Most cutaneous granulomas (75%) were non-necrotizing M-type or necrotizing M(n)-type, an epithelioid granuloma type with focally aggregated RVC^+^ M2 macrophages. The newly identified pattern, N-type of RuV-associated granulomas (25% of cutaneous granulomas), was characterized by a predominance of RVC^+^ neutrophils with most of them organized in a compact core with central necrosis. The fourth pattern of RuV-associated granulomas, DNI-type, which prevails in extracutaneous lesions, was a poorly organized granuloma characterized by diffuse infiltration of RVC^+^ neutrophils intermixed with monocytes, macrophages, and lymphocytes. Overlapping or even novel patterns may be identified as a number of characterized RuV-associated granulomas grows.

There was no association between specific granuloma patterns and the age of granuloma onset or the underlying error of immunity. The characteristic feature of all granuloma patterns was a substantial presence of T cells even though most individuals had low T-cell counts or defective T-cell cytotoxicity. Our analysis of the IHC images suggests that the spatial organization of RuV associated granulomas does not prevent close contact between T cells and infected macrophages and neutrophils (**Figure 3**). This contrasts with TB granulomas in which the spatial arrangement of cells limits the ability of T cells to reach infected macrophages at the granuloma centers (27). Other possibilities, such as T-cell exhaustion, limited generation and/or recruitment of RuV-specific T cells, and generation of RuV escape mutants, should be investigated to understand the mechanisms of RuV persistence.

Only one pattern of RuV-associated inflammation in IEI patients, M-type, has been previously described (16, 17, 25). The reasons why we were able to identify additional patterns in this study are the inclusion of individuals with diverse IEI types and the relatively large size of the patient cohort. In addition to the description of different granuloma patterns, this study also provides extensive clinical information on 28 patients and emphasizes the breadth of conditions where RuV should be considered as a potential etiology of granulomatous inflammation. On comparing with the published cases of RuV in patients with IEI, few commonalities emerge (**Table 3**). Most patients have T-cell quantitative and/or qualitative deficiency. Various types of CID prevail (71%) with ataxia telangiectasia being by far the most common (21/80, 26%) although this disease only constitutes 0.3%-8% in several national registries (28–33). Both CVID patients in our cohort had low CD3^+^ T cells and a phenotype supportive of abnormal T-cell function suggesting it was the T-cell defect rather than the humoral deficiency that contributed to RuV susceptibility. The genetic etiologies of the three HLH patients reported here, and 12 patients recently reported by Gross et al, which include the *RAB27A, UNC13D*, and *PRF1* genes, impacted CD8^+^ T-cell cytotoxicity (25). In addition, we report several previously undocumented conditions responsible for increased susceptibility to RuV -associated granulomas. DiGeorge syndrome is a recognized T-cell deficiency, related to impaired thymic T-cell production. The mechanism of susceptibility to RuV in patients with humoral immune deficiencies (P11 with IgG_2_ and IgA deficiencies) remains poorly understood as humoral immunity is not considered as a major mechanism of immune control and eradication of RuV in chronically infected individuals (20). P12 with XLA was being treated for his LGL with cyclophosphamide, a possible contributor. Additionally, maturation, recruitment, and function of macrophages and neutrophils depend on the Bruton’s tyrosine kinase, BTK (34), thus providing a potential mechanism for XLA patients. Although patients with *STAT1* loss-of-function mutations showed normal lymphocyte subsets and immunological reactions, they were highly susceptible to infections due to the lack of interferon responses, which were fundamental for the activation of the pathogen killing pathways in infected macrophages (35). Immunodeficiency in one patient (P6) was attributed to severe malnutrition, which often leads to immune dysfunction (36). Thus, susceptibility to RuV emergence from the tissue reservoir is likely more complex than merely having a T-cell defect. However, our data help to paint a somewhat clearer picture of the major susceptibility factors. The patients generally produced excessive amounts of RuV neutralizing antibodies and had some element of T-cell dysfunction but were generally not categorized as having SCID, which would have been a contraindication to immunization with live viral vaccines. In addition, secondary immunodeficiency due to immunosuppression for immune dysregulation or other etiology may play a role in triggering RuV emergence in adult patients who had developed granulomas many years after establishment of IEI diagnosis. Additional work to define innate, monocyte, T-cell, and antibody responses may reveal new insights into mechanisms of susceptibility to RuV persistence and granuloma formation in different tissues.

**Table 3:** RuV-associated inflammation in IEI patients described in this case series and reported in the literature.

	This case series n=28	Perelygina et al. (16) n=7	Neven et al. (17) n=9	Perelygina et al. (20)n=7	Buchbinder et al. (18)n=17	Gross et al. (25) n=12	Total n=80
IEI type	CID (19)	CID (7)	CID (8)	CID (7)	CID (16)	HLH 12/12	CID (57, 71%)
	HLH (3)		CVID (1)		Neutrophil (1)		HLH (15, 19%)
	CVID (2)						CVID (3, 4%)
	Innate (1)						Innate (1, 1%)
	XLA (1)						XLA (1, 1%)
	Humoral (1)						Humoral (1, 1%)
	Unknown (1)						Neutrophil (1, 1%)
							Undetermined (1, 1%)
Genetic cause	*ATM* (4)	*ATM* (4)	*ATM* (5)	*ATM* (3)	*ATM* (5)	*Rab27A* (7)	*ATM* (21)
	22q11.2 del (2)	No gene (3)	*RAG1*	No gene (3)	*NBN* (4)	*UNC13D* (3)	*RAB27A* (8)
	*RAG1* (2)		*RAG2*		*LIG4*	*PRF* (2)	*UNC13D* (5)
	*RAG2*		*PIK3CD*		*DCLRE1C*		*NBN* (5)
	*NBN*		No gene (1)		*RMRP*		*RAG1* (3)
	*TAP1*				*RFXANK*		*RAG2* (2)
	*TAP2*				*CXCR4*		*DCLRE1C* (2)
	*RAB27A*				*CORO1A*		*RMRP* (2)
	*ADA*				*IL2RG*		CORO1A
	*SLC7A7*				No gene (1)		*LIG4*
	*UNC13D*						*TAP1*
	*POLE*						*TAP2*
	*RMRP*						*ADA*
	*DCLRE1C*						*SLC7A7*
	*STAT1 (LOF)*						*POLE*
	*BTK*						*BTK*
	No gene (7)						*RFXANK*
							*CXCR4*
							*PRF (*2*)*
							*STAT1(LOF)*
							*IL2RG*
							*IKBKG*
							*PIK3CD*
RuV location	Skin (20)	Skin (7)	Skin (9)	Skin (7)	Skin (17)	Skin (9)	Skin (69, 86%)
	Bone marrow (6)	Bone (1)	Spleen (1)	Liver (2)		Lung (2)	Bone marrow (6, 8%)
	Brain (3)		Lymph node (1)	Bone (1)		Liver (1)	Liver (5, 6%)
	Liver (2)			Kidney (1)			Lung (4, 5%)
	GI tract (1)						Brain (3, 4%)
	Lung (2)						Lymph node (2, 3%)
	Pancreas (1)						Spleen (2, 3%)
	Lymph node (1)						Kidney (1, 1%)
	Spleen (1)						Bone (1, 1%)
	Joint (1)						Pancreas (1, 1%)
							Joint (1, 1%)
							GI tract (1, 1%)
Deceased	6	4	Not reported	3	3	Not reported	16/59 (27%)

Another important factor which allowed us to identify multiple RuV-associated patterns was testing of a broad array of tissues involved in inflammation. Patients with IEI and RuV-associated disease were primarily affected with cutaneous granulomas (86%). This was the original phenotype described (15, 16), and still constitutes the most common reason for referral for testing. There is likely some ascertainment bias because the skin is more accessible to biopsy than other organs. Nine extracutaneous tissues with RuV-associated granulomas were described in this case series including three newly recognized sites, i.e., bone marrow, brain, and GI tract (**Table 3**). The observations that RuV-associated granulomas can first emerge in extracutaneous body sites often distant from a vaccination site strongly argue against skin representing a unique RuV reservoir. There were clear differences in the RuV granuloma patterns in skin and extracutaneous locations. In skin, the focal aggregates of RuV infected macrophages or neutrophils most likely serve as the trigger for granuloma assembly around them. In extracutaneous locations, RuV may not be a major trigger of chronic inflammation but rather exacerbates it as DNI-type granulomas with abundant RVC^+^ neutrophils were typically seen near areas of RuV-unrelated inflammation, e.g., near RVC^-^ granulomas in P16 GI, P26 spleen, and P27 brain, or near the site of infection with other pathogens, e.g., CMV and *Histoplasma spp* in P16 GI.

This report is one of the few to describe therapeutic efforts to treat granulomas in IEI patients, which have not been standardized and were limited to small case series (18, 21, 23, 37). Unfortunately, the lack of an animal model for RuV persistent infections is impeding thorough investigations of antiviral strategies. The most common treatments were oral and topical steroids which were moderately effective in some patients in all case series. Consistent with other reports (18, 37), a broad-spectrum antiviral drug nitazoxanide provided only mild improvement. There have been responses in this case series to TNF-α and IL-1R inhibitors, both are known suppressors of myeloid-driven inflammation, suggesting that they offer some ability to resolve lesions in patients with granulomatous inflammation dominated by chronic neutrophil infiltration. However, there were also patients in this series where these same therapies failed. HCT was the most successful treatment, which was performed in seven patients in this series with resolution of the lesions in five successfully transplanted cases. Lacking direct anti-viral agents that target RuV, there is clearly a need for improved management of this subset of patients with IEI.

The present study opens an intriguing possibility that the bone marrow neutrophils or their myeloid precursors provide a niche for long-term RuV persistence. There was notable presence of RVC^+^ neutrophils in both normal and abnormal bone marrow samples of 6/7 (86%) patients. The observation that the lesions clinically improve when patients are being conditioned for bone marrow transplantation, even before they receive their graft, supports the importance of the bone marrow as an RuV reservoir and the neutrophils as their vehicle. Interestingly, live RuV was often recovered from bone marrow aspirates in infants with congenital rubella syndrome, although RuV target cells in bone marrow have never been determined (38). RuV persistence in bone marrow neutrophils may impair their differentiation into mature neutrophils as well as alter their functions. Several other viruses have been implicated in bone marrow suppression while persisting in erythroid precursors (parvovirus 19, dengue) or bone marrow stromal cells (respiratory syncytial virus) (39–41).

**Figure 9:**
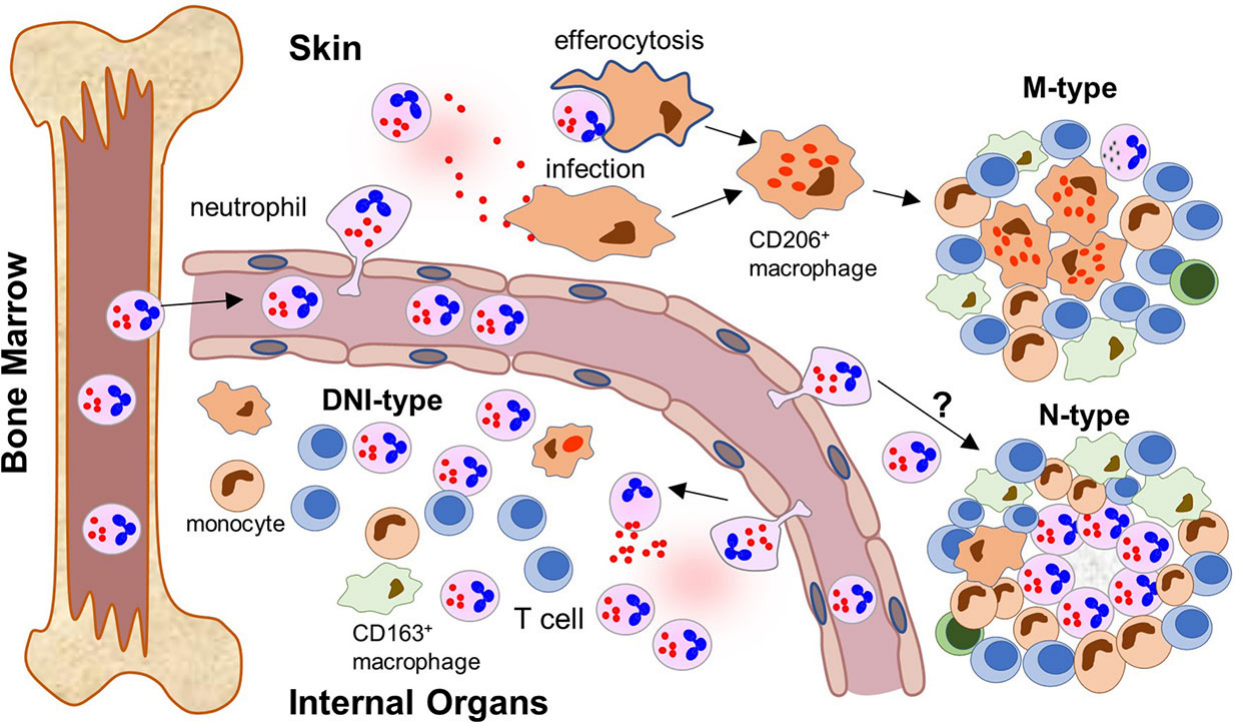
Neutrophil recruitment to inflamed tissues and formation of different patterns of RuV associated inflammation (a proposed model). After maturation, RuV infected mature neutrophils leave the bone marrow where RuV subclinically persisted in myeloblasts before weakening of the immune control mechanisms. The circulating neutrophils recognize signs of ongoing inflammation and migrate into the tissue. The location of the inflammatory signal and microenvironment at the site influence the formation of a particular pattern of RuV-associated granulomas. In skin, tissue macrophages become infected with RuV either by extracellular virions released by neutrophils or after ingesting infected neutrophils. They subsequently differentiate into epithelioid CD206^+^ macrophages. T cells, which are recruited to the infected tissues but cannot eradicate RuV infection, form a structure of a mature granuloma around the RuV^+^ macrophage core together with migrating monocytes and macrophages. Presence of B lymphocytes and neutrophils in the mature M-type RuV-associated granulomas is negligible. Some individuals develop N-type necrotizing granulomas with RuV neutrophil cores, but the underlying mechanism is unclear. Influx of RuV infected neutrophils into inflamed internal organs and tissues attracts immune cells of the innate and adaptive immunity but does not result in the formation of compact granuloma structures. Instead, diffuse inflammation pattern dominated by RuV positive neutrophils intermixed with other immune cells (DNI-type) is formed.

The presented data support an evolving model where RuV persists subclinically in neutrophil precursor cells in the bone marrow and emerges with loss or diminution of especially T-cell immune control, similar to JC virus (42). We propose that RuV uses mature neutrophils as a vehicle for broad tissue dissemination (**Figure 9**). RuV^+^ neutrophils are recruited to the sites of inflammation (sterile or not sterile) where they amplify the local inflammation and contribute to the development of chronic RuV-associated inflammation. Which inflammatory pattern develops, M-type, M(n)-type, N-type, or DNI-type, likely depends on the microenvironment at the site of the initial inflammation and the underlying IEI. We hypothesize that RuV infection of macrophages is an intermediate step to M-type and M(n)-type granulomas. There are two possible mechanisms for macrophage infection. As we have shown, viral capsid and, likely, RuV virions are localized in primary neutrophil granules and could be released into extracellular space during the degranulation process. These extracellular RuV can subsequently infect macrophages and establish persistent infection. It is also possible that RuV can infect macrophages following efferocytosis of apoptotic infected neutrophils, a mechanism described for *Leishmania* and other parasitic infections (43). Persistently RuV-infected macrophages in the absence of functional T-cell immunity can subsequently trigger formation of M-type or M(n)-type granulomas. In addition to the proposed role in the initiation of M-type granulomas, uninfected neutrophils in a subgroup of patients can also be mobilized within M(n)-type necrotizing RVC^+^ granuloma cores leading to central necrosis. Signals triggering the development of M(n)-type necrotizing inflammation are currently unknown.

The mechanisms of development of N-type and DNI-type granulomas are less clear. Neutrophils are short-lived cells and die by apoptosis within one day after entering tissues. Apoptosis reduces the number of neutrophils in tissue and sends signals to stop neutrophil influx as well as to induce re-programming of macrophages to an anti-inflammatory phenotype (44). Although it has not been demonstrated for RuV, several other viral pathogens have been shown to manipulate apoptotic and survival pathways in neutrophils for their advantage (43). Given that many RVC^+^ neutrophils accumulate in inflamed internal organs or form large N-type cutaneous granulomas, RuV may also interfere with apoptosis and extend the neutrophil lifespan. Excessive neutrophil accumulation and delayed apoptosis usually leads to hyperinflammation and, instead of apoptosis, results in necrotic cell death followed by massive release of proteolytic and highly cytotoxic granular proteins, thus contributing to chronic inflammation and organ damage (43, 44). Resolving neutrophilic inflammation, therefore, may provide clinical benefit for patients with N-type and DNI-type RuV-associated granulomas. Rare clinical responses to steroids and TNF-α inhibitors suggest that there is an early stage where this might be possible (**Table 3**). Thus, a better understanding of the role of neutrophils in RuV associated granuloma pathologies is critical to improving treatment options of this complication in immunodeficient patients.

## Data Availability Statement

The original contributions presented in the study are included in the article/**Supplementary Material**, further inquiries can be directed to the corresponding author/s.

## Author Contributions

LP, JI, KS, and FH designed the study. LP, RF, EA, MC, and LH analyzed samples provided by all other authors. Acquisition and interpretation of clinical data: All authors. Drafting of the manuscript: LP, KES, and FH. Critical revision of the manuscript: All authors. All authors contributed to the article and approved the submitted version.

## Funding

This work was supported by core funding from the Centers for Disease Control and Prevention and by a grant to KS from the National Institute of Health (R21-AI130967-01A1). FH was funded by the Else Kröner-Fresenius Stiftung (EKFS, 2017_A110), and the German Federal Ministry of Education and Research (BMBF, 01GM1910C). TA was funded by a grant from the National Institute of Health (R01AI141877) and State of Alabama SCID Newborn Screening Contract.

## Author Disclaimer

The findings and conclusions in this report are those of the authors and do not necessarily represent the official position of the United States Centers for Disease Control and Prevention.

## Conflict of Interest

MA is employed by Sidra Medicine and Hamad Medical Corporation, Qatar. HB is employed by Labor Berlin GmbH, Germany. JH received grants from Immune Deficiency Foundation, the US immunodeficiency network, Chao-physician Scientist award, the Texas Medical Center Digestive Diseases Center and the Jeffrey Modell Foundation. JH received honorarium, consultation fees from Horizon, Pharming, Baxalta, CSL Behring, the National guard, and Al-Faisal University Hospital. TPA received consultation fees from Horizon, Pharming, CSL Behring.

The remaining authors declare that the research was conducted in the absence of any commercial or financial relationships that could be construed as a potential conflict of interest.

## Publisher’s Note

All claims expressed in this article are solely those of the authors and do not necessarily represent those of their affiliated organizations, or those of the publisher, the editors and the reviewers. Any product that may be evaluated in this article, or claim that may be made by its manufacturer, is not guaranteed or endorsed by the publisher.
