# Supplementary material for: Rubella Virus Infected Macrophages and Neutrophils Define Patterns of Granulomatous Inflammation in Inborn and Acquired Errors of Immunity
Source: Front Immunol. 2021 Dec 20;12:796065. doi: 10.3389/fimmu.2021.796065 (PMC8728873; doi:10.3389/fimmu.2021.796065)
Supplement: Supplementary file 1 [file DataSheet_1.pdf]

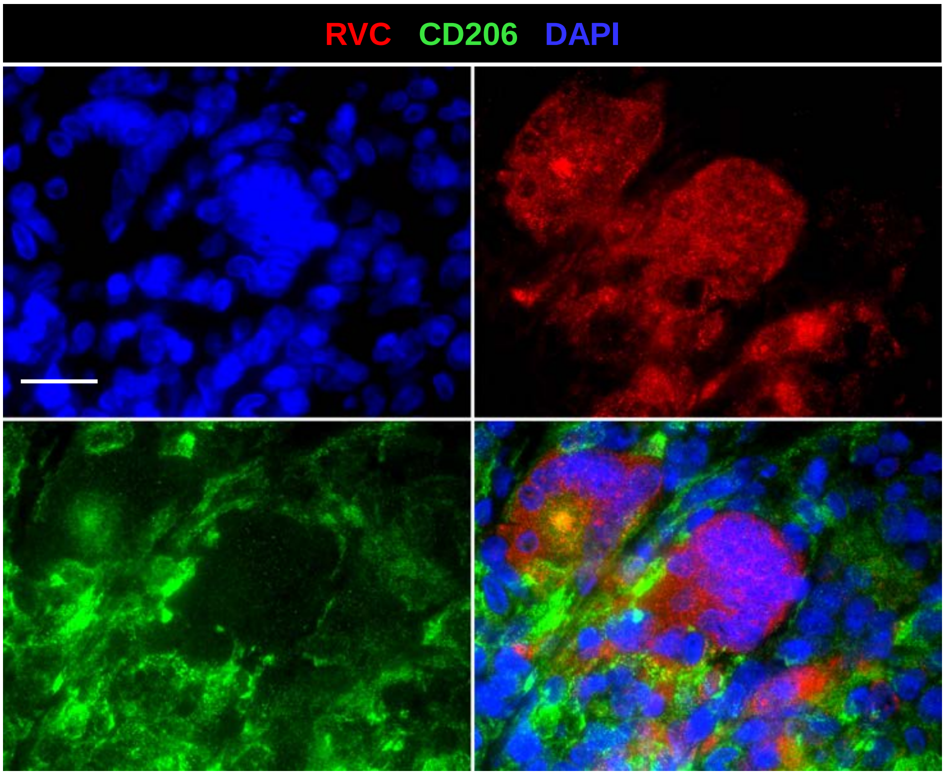

Supplementary Figure 1 Langhans giant cells in cutaneous granuloma. Double immunofluorescent staining for RVC and CD206 shows the presence of RVC^{+} Langhans giant cells (macrophage syncytia) in P16 cutaneous granuloma. Scale bar: 20 µm.
