# Supplementary material for: Rubella Virus Infected Macrophages and Neutrophils Define Patterns of Granulomatous Inflammation in Inborn and Acquired Errors of Immunity
Source: Front Immunol. 2021 Dec 20;12:796065. doi: 10.3389/fimmu.2021.796065 (PMC8728873; doi:10.3389/fimmu.2021.796065)
Supplement: Supplementary file 2 [file DataSheet_2.pdf]

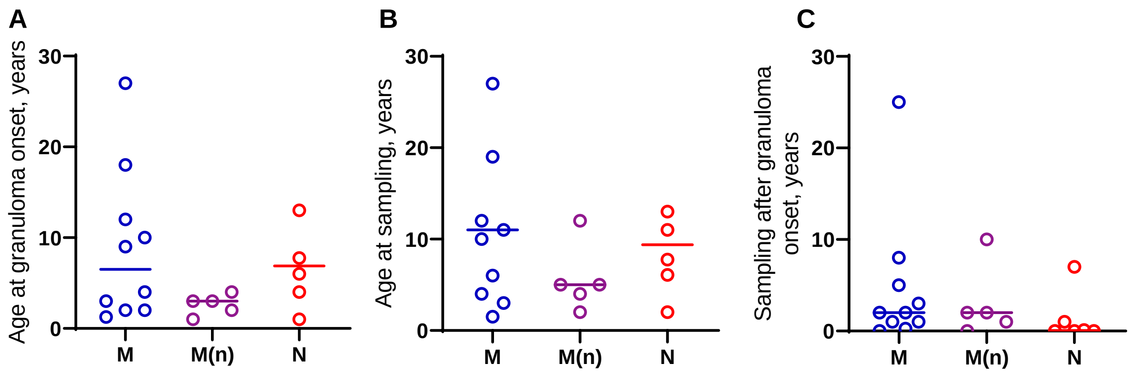

Supplementary Figure 2 Characteristics of different patterns of cutaneous granulomas. Age of the patients at the granuloma onset (**A**). Age at granuloma sampling (**B**). Time between sampling and granuloma onset (**C**). Horizontal lines indicate the median values.
