# Supplementary material for: Rubella Virus Infected Macrophages and Neutrophils Define Patterns of Granulomatous Inflammation in Inborn and Acquired Errors of Immunity
Source: Front Immunol. 2021 Dec 20;12:796065. doi: 10.3389/fimmu.2021.796065 (PMC8728873; doi:10.3389/fimmu.2021.796065)
Supplement: Supplementary file 3 [file DataSheet_3.pdf]

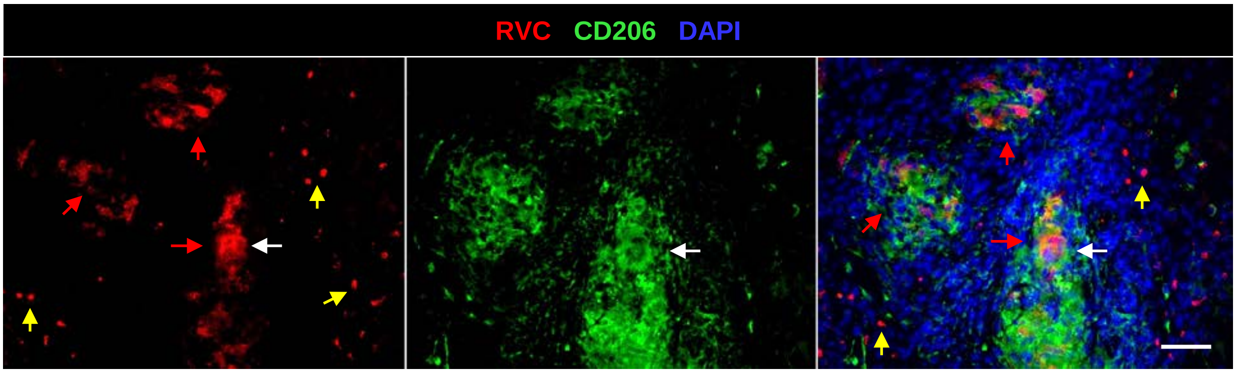

Supplementary Figure 3 RuV in liver granuloma. Histological double immunofluorescent staining for RVC and CD206 shows $RVC^{+}CD206^{+}$ M2 macrophages (the red arrows) in the center of M-type granuloma in P1 liver. The yellow arrows indicate RVC^{+} neutrophils on the granuloma periphery. The white arrows indicate RVC Langhans giant cell. Scale bar: 20 µm.
