# Supplementary material for: Rubella Virus Infected Macrophages and Neutrophils Define Patterns of Granulomatous Inflammation in Inborn and Acquired Errors of Immunity
Source: Front Immunol. 2021 Dec 20;12:796065. doi: 10.3389/fimmu.2021.796065 (PMC8728873; doi:10.3389/fimmu.2021.796065)
Supplement: Supplementary file 4 [file DataSheet_4.pdf]

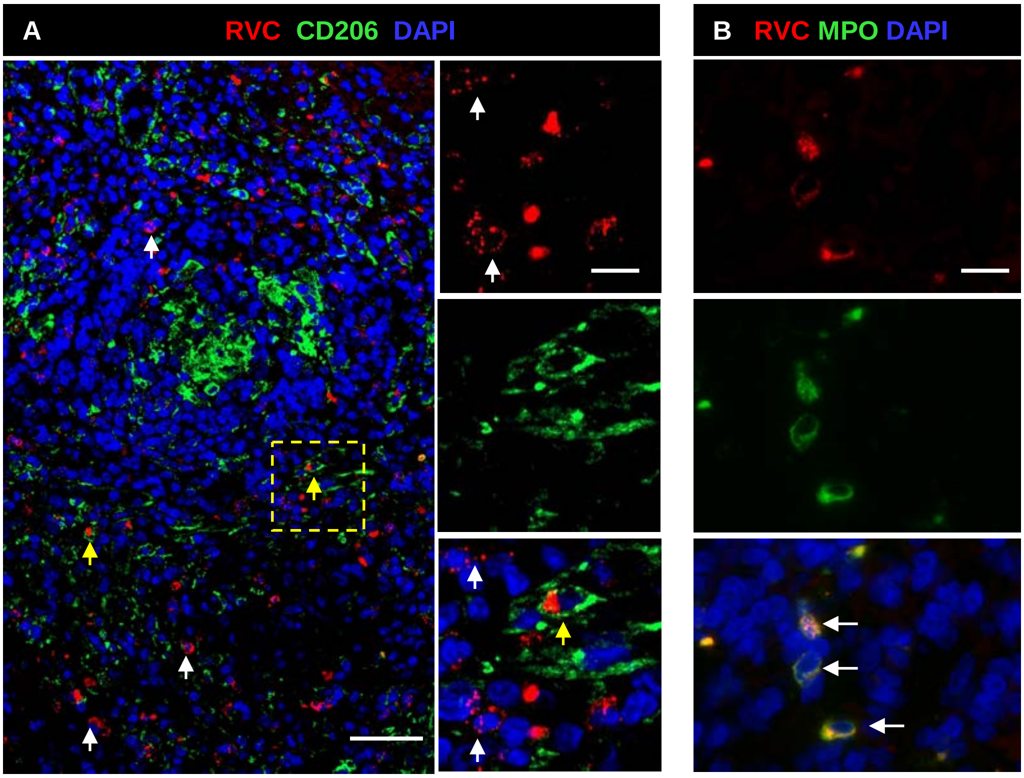

Supplementary Figure 4 RuV in spleen biopsy. Histological double immunofluorescent staining for RVC and either CD206 (**A**) or MPO (**B**) showing $RVC^{+}MPO^{+}$ neutrophils (white arrows) and $RVC^{+}CD206^{+}$ macrophages (yellow arrows) at the periphery of RVC^{-} granuloma in P26 inflamed spleen. Scale bars: 50 µm (**A**) and 10 µm (inlet and **B**).
