# Supplementary material for: Rubella Virus Infected Macrophages and Neutrophils Define Patterns of Granulomatous Inflammation in Inborn and Acquired Errors of Immunity
Source: Front Immunol. 2021 Dec 20;12:796065. doi: 10.3389/fimmu.2021.796065 (PMC8728873; doi:10.3389/fimmu.2021.796065)
Supplement: Supplementary file 5 [file DataSheet_5.pdf]

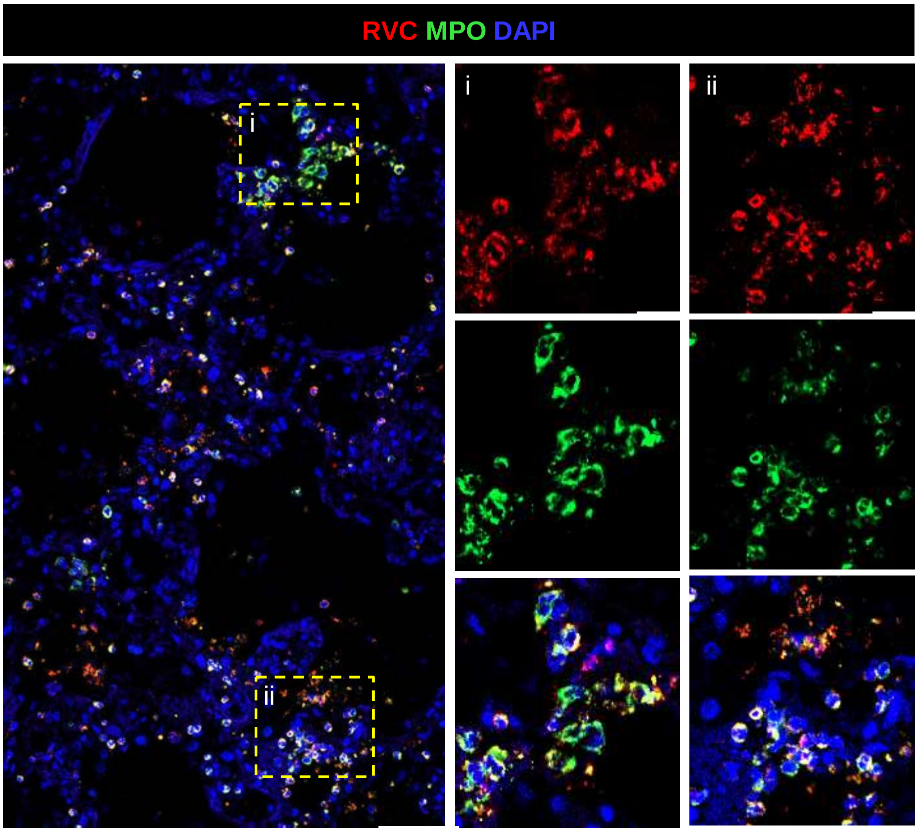

Supplementary Figure 5 RuV in lung biopsy. Histological double immunofluorescent staining for RVC and MPO showing numerous RVC^{+}MPO^{+} neutrophils in P24 inflamed lungs. Scale bars: 100 µm and 20 µm (inlets i and ii).
