# Supplementary material for: Rubella Virus Infected Macrophages and Neutrophils Define Patterns of Granulomatous Inflammation in Inborn and Acquired Errors of Immunity
Source: Front Immunol. 2021 Dec 20;12:796065. doi: 10.3389/fimmu.2021.796065 (PMC8728873; doi:10.3389/fimmu.2021.796065)
Supplement: Supplementary file 6 [file DataSheet_6.pdf]

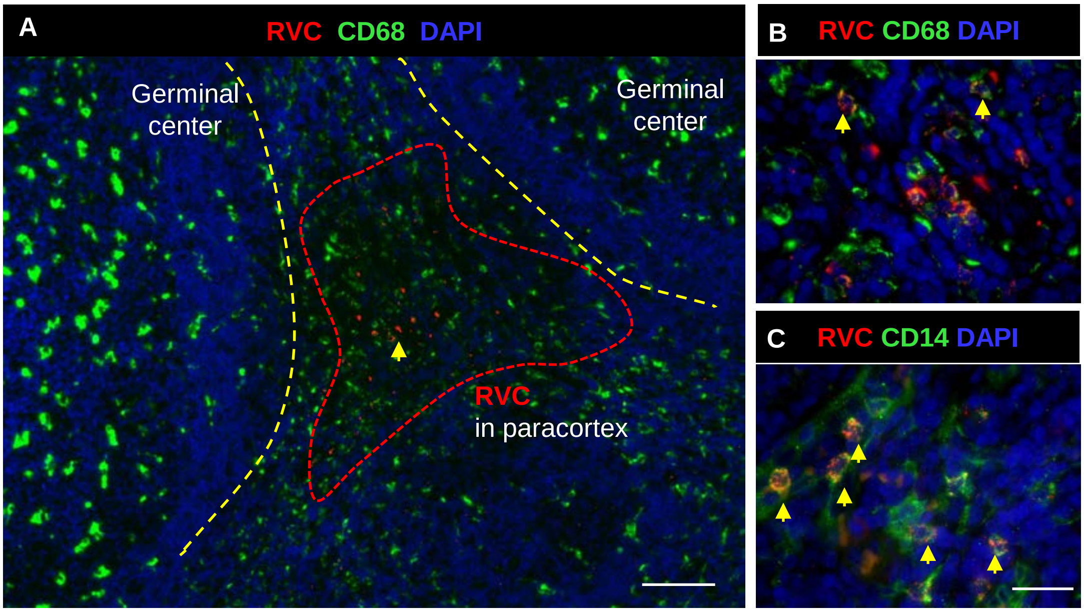

Supplementary Figure 6 RuV in lymph node biopsy. Histological double immunofluorescent staining for RVC (**A-C**) and either CD68 (**A, B**) or CD14 (**C**) shows that RVC in the paracortex of P10 axillary lymph node localized mainly in $CD14^+$ monocytes and sporadically in $CD68^+$ macrophages. Yellow arrows indicate RVC^+ cells. Scale bars: 100 µM (**A**) and 20 µM (**B, C**).
